# Evaluation of the effectiveness of the use of exosomes in the regulation of the mitochondrial membrane potential of frozen/thawed spermatozoa

**DOI:** 10.1371/journal.pone.0303479

**Published:** 2024-07-03

**Authors:** Alicja Kowalczyk, Władysław Kordan

**Affiliations:** 1 Department of Environment Hygiene and Animal Welfare, Wrocław University of Environmental and Life Sciences, Wrocław, Poland; 2 Department of Animal Biochemistry and Biotechnology, University of Warmia and Mazury, Olsztyn, Poland; Jawaharlal Nehru Technological University Hyderabad, INDIA

## Abstract

Numerous studies confirm the involvement of extracellular vesicles (EVs) in the regulation of physiological processes of mammalian sperm cells. It has been proven that they take part in the processes of capacitation, acrosonmal reaction, and anti-oxidation. Despite growing interest in the biomedical potential (including the search for new reproductive biomarkers) of EVs, the role of extracellular seminal vesicles in maintaining semen quality during cryopreservation has not yet been established. Therefore, the objective of this experiment was to evaluate the effectiveness of the use in the regulation of the mitochondrial membrane potential of bovine sperm and to explain the mechanisms of EV action during cell cryopreservation. Exosomes were isolated from bull semen plasma, measured, and used for extender supplementation. Semen samples were collected from Simmental bulls, diluted, and pre-evaluated. Then they were divided into equal fractions that did not contain EVs or were supplemented with 0.75; 1.5 and 2.25 mg/ml of EVs. The test samples were frozen/thawed and the mitochondrial membrane potential, DNA integrity, and viability were evaluated. EVs have been established to have a positive effect on cryopreserved sperm structures. The most favourable level of EVs was 1.5 mg / ml, which can be successfully to improve cell cryostability during freezing/thawing. In this study, exosomes isolated from the sperm plasma and supplemented with a concentrated dose in the extender for sperm freezing were shown to significantly improve cryostability of cells by supporting the potentials of the mitochondrial membrane and protecting the cytoplasmic membrane of spermatozoa.

## Introduction

The nomenclature of extracellular vesicles (EVs) refers to a heterogeneous population that varies in size, biogenesis, composition, and function secreted into the extracellular space by various cells [1]. They are commonly classified according to class, the most important of which are exosomes (40–160 nm), microvesicles (MV, 150–1000 nm), and apoptotic bodies (>1000 nm) [2]. Interestingly, numerous studies confirm the involvement of EVs in the regulation of physiological processes of mammalian sperm cells. It has been proven that they participate in the processes of capacitation, acrosonmal reaction, and anti-oxidation [3, 4, 32].

As we have written previously [5], seminal plasma exosomes play a key role, among others, in inducing regulation of sperm motility [29, 30] and protecting sperm in the environment of the female reproductive system by modulating their activity in response to the acidic environment of the vagina [6].

Mitochondria are important cellular organelles involved in spermatogenesis, sperm capacitation, viability, and fertilisation [7, 31]. They are involved in energy metabolism, the tricarboxylic acid cycle, oxidative phosphorylation, and calcium ion [7, 8]. In addition, these structures are the main site for the formation of intracellular reactive oxygen [9]. Symptoms within these organelles result in disorders in spermatogenesis [7]. Mitochondrial dysfunction in sperm is mainly caused by oxidative stress, which in turn results in impaired ATP transport and, consequently, reduced sperm motility [8]. The mitochondrial membrane potential has been shown to be positively correlated with the viability and sperm fertilization rate of the sperm [7].

Despite growing interest in the biomedical potential (including the search for new reproductive biomarkers) of EV, the role of extracellular seminal vesicles in maintaining semen quality during cryopreservation has not yet been established. Furthermore, the basic mechanisms of the interaction of plasma sperm exosomes with spermatozoa are poorly understood.

Therefore, the objective of this experiment was to evaluate the efficacy of the use in the regulation of the mitochondrial membrane potential of bovine spermatozoa and to explain the mechanisms of action of the action during cell cryopreservation. An additional objective was to investigate the relationship between the maintenance of the REDOX balance in semen and the potential of the mitochondrial membrane and the viability of cryopreserved spermatozoa in the presence of different concentrations of EVs.

## Materials and methods

### Semen processing

The experiment was carried out as part of routine procedures used in the Reproduction Centre with full respect for the animals. According to the applicable law, it did not require the consent of the Local Ethics Committee was not required.

These experiments were carried out at the Malopolska Biotechnik Centre (Krasne, Poland). In this experiment, biological material (semen) from 13 healthy Simmental bulls was collected once a week at 7 am using an artificial vagina (two ejaculates from each male). The animals included in the study were of similar age (3 ± 0.5 years), kept and fed identically (a mixture of farm fodder: haylage, maize silage, apple pomace, basic mineral, and vitamin premix), with constant access to water.

The collected ejaculate samples were stored in a water bath at 37°C, where they awaited preliminary quality analysis and dilution. A commercially available extender (OptiXcell®, IMV Technologies, L’aigle, France) was used to obtain a final sperm concentration of 150 × 10^6^ spermatozoa/ml. The semen prepared in this way was then subjected to cell membrane integrity analysis, and samples containing at least 60% live spermatozoa were qualified for further procedures. After a positive evaluation, semen samples were pooled to eliminate individual differences and divided into equal fractions.

The first fraction was left for the control group (without EVs), and the next was added in succession 0.75; 1.5; and 2.25 mg/ml of EVs. Then all samples were incubated at 37.5°C for 45 min. After that, semen was automatically packed (Bloc Machine FIN, IS 4, France) and equilibrated for 1.5 h at 4° C. After that, semen was frozen in liquid nitrogen vapour using a computer-controlled automatic freezer (IMV Technologies). After week, the straws were thawed in a water bath at 38°C for 20 s and then were examined [10].

### Exosome isolation

To isolate exsomes from bull seminal plasma, a differential centrifugation method in combination with serial ultracentrifugation was used according to the protocol of Murdic et al. [11]. Plasma exosomes were isolated as previously described [12]. The sperm-rich fraction was subjected to sequential centrifugation (1000 x g for 15 min at 24°C and 10,000 x g for 30 min at 4°C and 20,000 x g for 30 min at 4°C) to obtain seminal plasma free of spermatozoa and residues cells. The pellet was used to separate the microbubbles and the supernatants were filtered through 0.45 and 0.22 μm filters (Millipore, MA, USA) and ultracentrifuged at 100,000 x g for 1 hour at 4°C using a Beckman Coulter L5-65 ultracentrifuge. The resulting pellets were washed twice with phosphate buffered saline (PBS) and repelled by ultracentrifugation. After resuspension in 2 ml of this buffer, the vesicles were purified by gel filtration on a Sephadex G-200 column (210–20 mm) pre-equilibrated with the same buffer. The volume of the void, which contained the exosomes, was centrifuged at 120 000 g for 1 h at 4° C and the pellet was resuspended in PBS [32].

### Quantification and determination of exosome size

The nanoparticle tracking analyse was made using the Nanosight LM-10 instrument (Nanosight) as described by the manufacturer and used to measure the concentration and size distribution of isolated exosomes. In brief, exosome samples were vortexed and serially diluted to a final dilution of 1:8000–1:80 000 in filtered molecular grade H_2_O. Each sample analysis was conducted for 60 s using Nanosight automatic analysis settings. The samples were evaluated in triplicate and the concentration values were averaged [13, 32].

### Sperm mitochondrial membrane potential

The JC-1 probe (5,5’,6,6’-tetrachloro-1,1′,3,3′-tetraethyl- benzimidazolylcarbocyanine chloride, (Invitrogen) was used to determine the mitochondrial membrane potential and was divided according to emission intensity: a) with green fluorescence, low mitochondrial potentials (LMP) and medium mitochondrial potentials were determined; b) samples emitting red-orange fluorescence were labelled as cells with high mitochondrial potential (HMP). Flow cytometry (CytoFlex Beckman Coulter, B3-R1-V0, China) was used to determine this parameter by analysing 200,000 cells diluted in SP-Talp and stained with JC-1 (76.5 μmol/L in DMSO) 10 minutes after sample preparation. Slight modifications to the protocol have been applied to the previously described procedure [10].

### Sperm viability

Semen was thawed under controlled conditions (37°C for 20 s), then 50 μl of sperm, 940 μl of NaCl (0.9%) and 5 μl of SYBR14 (double staining of SYBR-14 with propidium iodide (L-7011 LIVE/DEAD Sperm Viability Kit; Invitrogen, Molecular Probes)) and thoroughly mixed, then incubated at 36°C for 10 minutes. The prepared samples were mixed again with 5μl PI and incubated in the dark for another 3 minutes. The test was performed using flow cytometry (CytoFlex Beckman Coulter, B3-R1-V0, China) [10].

### Sperm DNA integrity

In situ evaluation of chromatin sensitivity to acid-induced denaturation was used to analyse cell DNA integrity. Flow cytometry combined with the sperm chromatin structure assay (SCSA) was used to quantify the chromatin instability (CytoFlex Beckman Coulter, B3-R1-V0, China). Initially, semen was thawed at 26 for 30 seconds, then 13 μl was dispensed into a glass tube and thoroughly mixed with 487 ml of NaCl (0.9%) and placed on ice. To a second tube placed on ice, 50 μl of this mixture was measured and 100 ml of acidic detergent solution (0.08 M HCl, 0.15 M NaCl, 0.1% v/v Triton X-100, pH 1.2) was added and incubated for 30 seconds in the dark. Na_2_HPO_4_ per ml of citrate buffer (0.037 M citric acid, 0.126 M, 1.1 mM disodium EDTA, 0.15 M NaCl, pH 6.0) was added to the prepared mixture and incubated on ice for another 3 minutes, followed by cytometric analysis [14].

### Sperm biochemical assays

Semen samples were centrifuged at 750 rpm for 10 min to remove the supernatants, obtained cell pellets were washed three times with PBS and then incubated with 0.2% Triton X-100 on ice for 20 min. Superoxide dismutase (SOD) and catalase (CAT) activities were determined by spectrophotometry, by commercially available enzyme activity detection kits (Jiancheng Institute of Bioengineering), and the malondialdehyde (MDA) by thiobaby Tauric acid (TBA) assay using a chemical reaction kit (Built Bioengineering Institute) following the manufacturer’s instructions, as previously described [14].

### Statistical analysis

Analysis of variance (ANOVA) was used to assess differences between seminal plasma exosomes supplementation on all characteristics of spermatozoa. When the F ratio was significant (P< 0.01), Duncan’s multiple range test was used to compare treatment means. Statistical analysis of the results was performed with Statistica 13.0 (StatSoft, Poland).

## Results

**Fig 1:**
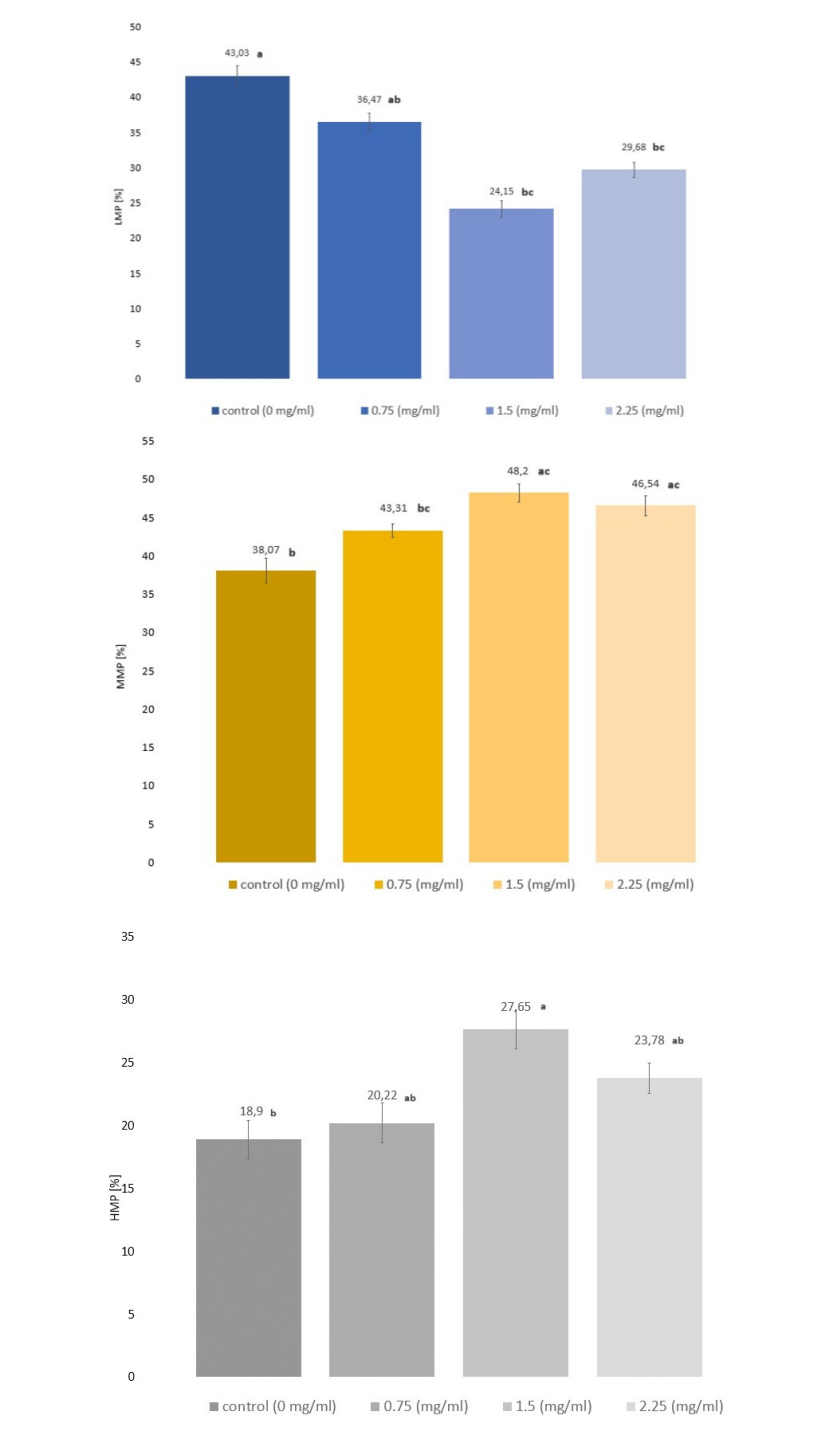
Mitochondrial membrane potential in frozen/thawed spermatozoa with exosomes addition. A) Low Mitochondrial Potential, B) Medium Mitochondrial Potential, C) High Mitochondrial Potential. Explanations: a, b—the means with different letters in the same column differ significantly at P < 0.01. The values are expressed as mean ± SD. Description: LMP–low mitochondrial membrane potential. MMP–medium mitochondrial membrane potential. HMP–high mitochondrial membrane potential.

The results of the analysis of the mitochondrial membrane potential of frozen/thawed spermatozoa cryopreserved in an extender supplemented with various concentrations of EVs are presented below (Fig 1A–1C).

The analysis (Fig 1C) shows that the percentage of sperm with the highest HMP was recorded in the two groups receiving 1.5 mg/ml (27.65%) and 2.25 mg/ml (23.78%) EVs, while the lowest values were observed in the two remaining groups control (18.9%) and 0.75 mg/ml (20.22%). HMP results between the control group and the two test groups with the highest EVs concentration (1.5 mg/ml and 2.25 mg/ml) differed significantly at the P <0.01 level. In terms of MMP (Fig 1B), significantly higher (P <0.01) values of this parameter (P <0.01) were observed for the two research groups that received the highest concentration of EVs, by 10.13% (1.5 mg/ml) and 8.47% (2.25 mh/ml) respectively. The highest LMP (Fig 1A) was observed in the control group (without EVs) where this parameter reached an average value of 43.03%. This value was significantly different (P< 0.01) from the other two test groups that received 1.5 and 2.25 mg / ml EV, where the LMP value was also the lowest of all groups tested. The analysis shows that the sperm stored in the extender supplemented with EVs and subjected to the cryopreservation process have better mitochondrial membrane potential parameters than the control group.

**Fig 2:**
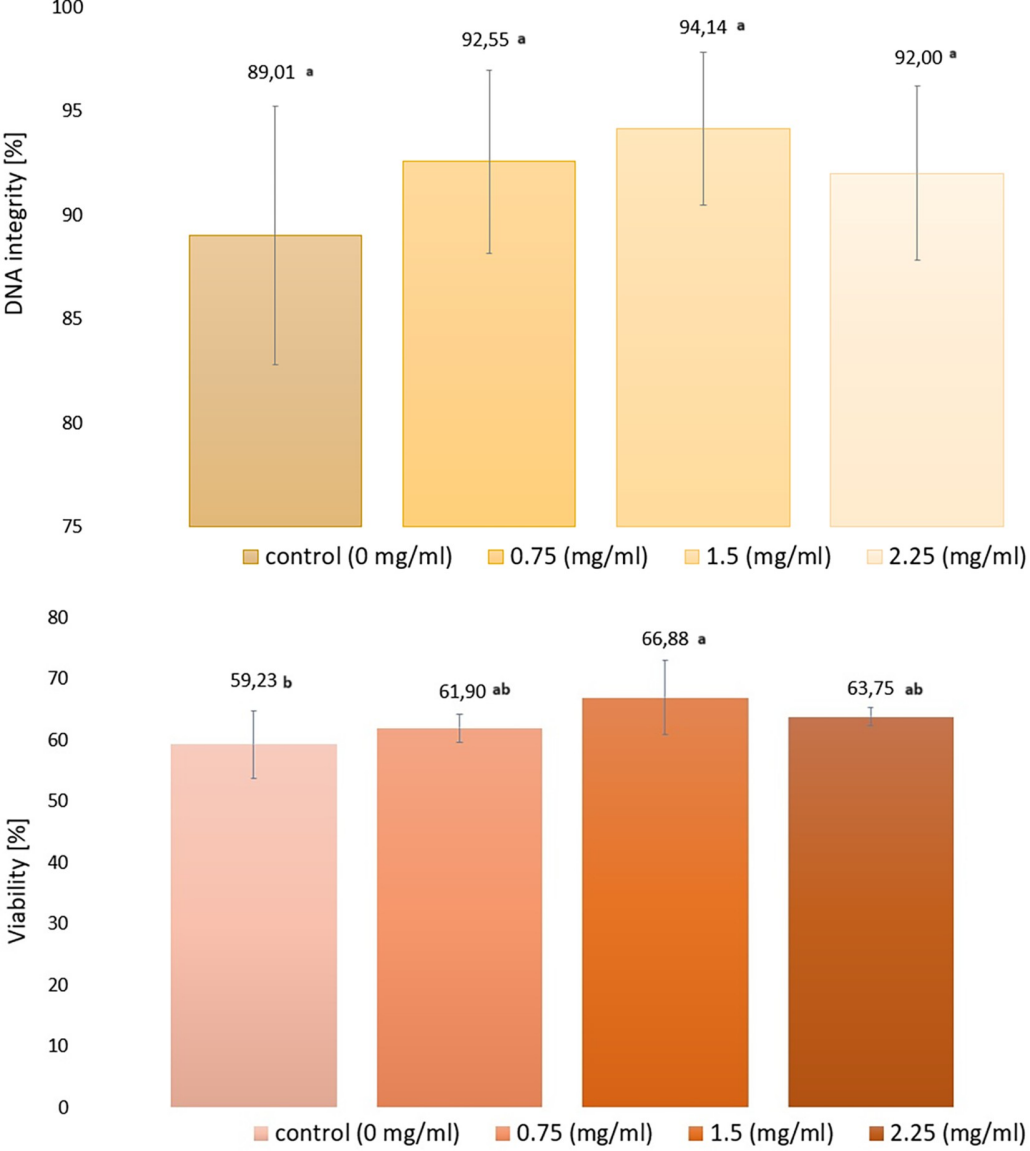
DNA integrity and viability of frozen/thawed spermatozoa in extender supplemented with exosomes. A) DNA integrity, B) Viability, Explanations: a, b—the means with different letters in the same column differ significantly at P < 0.01. The values are expressed as mean ± SD.

The results of the analysis of DNA integrity and viability of frozen / thawed spermatozoa with the addition of EVs are presented in Fig 2A and 2B).

In terms of DNA integrity (Fig 2A), no significant differences were observed between the tested samples (P <0.01), however, there was an increase in the percentage of sperm with intact DNA in the test groups (0.75; 1.5; 2.25) by 3.54; 5.13 and 2.99% over the control group (89.01%). A significant increase (P <0.01) in sperm with intact of cytoplasmic membrane (live sperm) (Fig 2B) was observed in the group receiving 1.5mg/ml of EV, where the percentage was 66.88% and 7.65% higher than in the control group (59.23%). The remaining studied groups did not differ significantly; however, a clear upward trend was observed in terms of the analysed parameter. In the groups receiving 0.75 and 2.25 mg/ml EVs, sperm viability was higher by 2.67% and 4.52%, respectively, compared to the control group without the addition of EVs.

**Fig 3:**
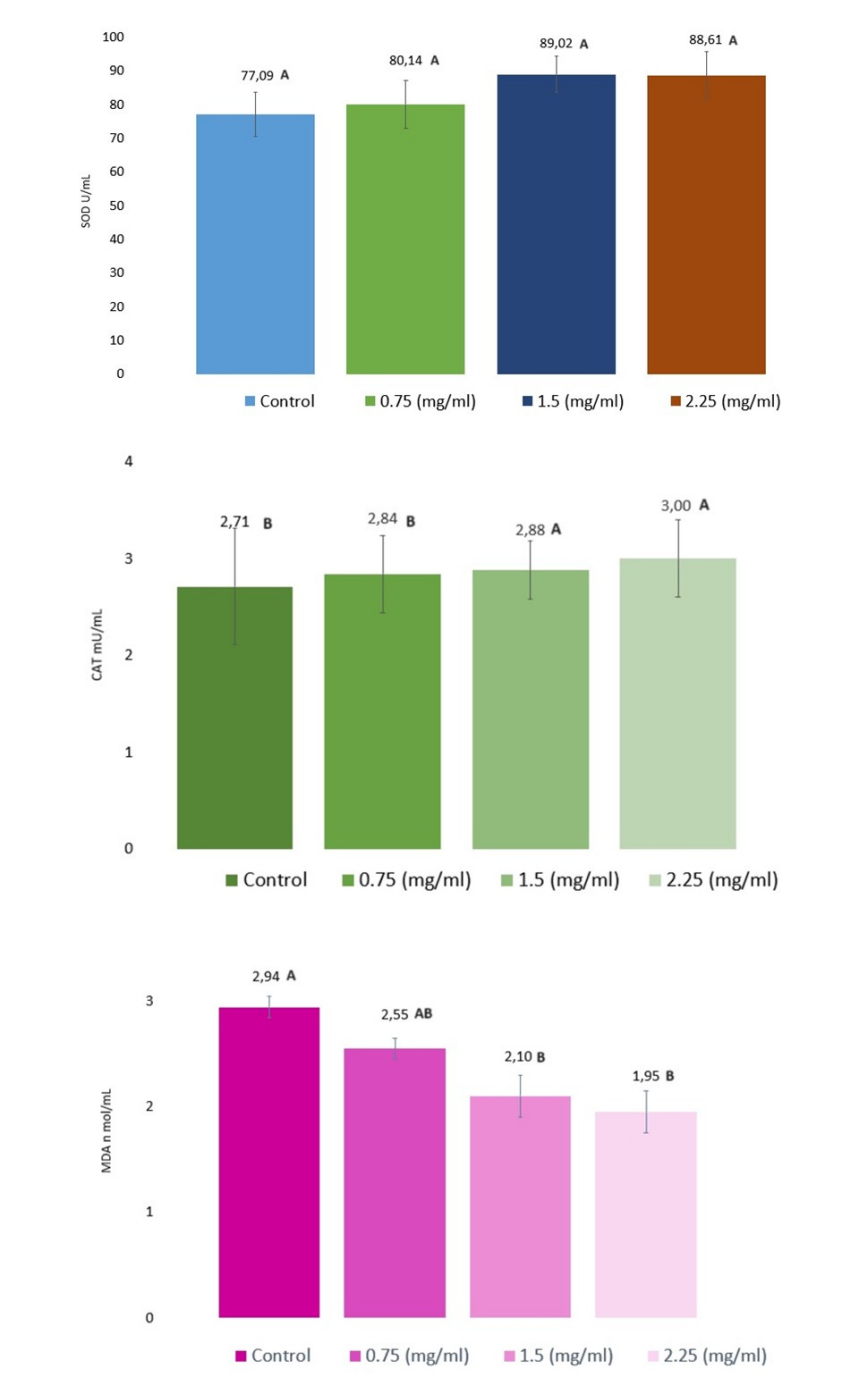
Antioxidant evaluation of frozen/thawed spermatozoa in extender supplemented of exosomes. A) SOD, B) CAT, C) MDA, Explanations: A, B—the means with different letters differ significantly at P < 0.001. The values are expressed as mean ± SD.

Analysing the results in the field of antioxidant enzyme activity (Fig 3A–3C), it can be seen that no significant fluctuations in activity were observed in the SOD range. The highest SOD activity was observed in the group with the addition of 1.5 mg/ml EVs, which was higher by 11.52 U/ml compared to the control (77 U/ml). The effectiveness of EVs in the remaining tested groups, i.e. 0.75 and 2.25, was higher, because the SOD activity results were 3.05 and 11.52 mg/ml higher, respectively, than in the control group. A clear upward trend in this parameter was observed in groups supplemented with EVs. However, both CAT and MDA in the 1.5 and 2.25 mg/ml EVs groups improved significantly (P <0.001). The highest CAT score was obtained in the group supplemented with 2.25 mg/ml EVs where this value was 3.0 mU/ml (control group 2.71 mU/ml). The highest MDA activity was present in the control group, where this value reached 2.94 n mol/mL. In the remaining groups, a significant decrease in this enzyme was seen by 0.39, 0.84, 0.99 n mol/mL in the groups receiving 0.75, 1.5, 2.25 mg / ml of EVs, respectively.

## Discussion

The purpose of this experiment was to evaluate exosome effectiveness of the use in the regulation of the mitochondrial membrane potential of frozen/thawed bovine sperm.

Exosomes are small extracellular vesicles secreted by various cell types, including sperm cells. They are typically spherical in shape and have a lipid bilayer membrane. Exosomes contain various bioactive molecules, including proteins, lipids, nucleic acids (such as RNA and DNA), and metabolites. These vesicles play an important role in intercellular communication by transferring their cargo to recipient cells, thus influencing various physiological and pathological processes. [15–17].

Despite research efforts, the sperm cryopreservation still leads to multiple damage related to thermal, osmotic and dehydration shocks, ice crystal formation, and elevated levels of reactive oxygen species (ROS) [18–21]. Furthermore, the presence of high levels of polyunsaturated fatty acids in the sperm membrane and low levels of antioxidant components in the limited cytoplasm make them more susceptible to cryodamage [22, 23]. In a study by Leite et al. [23] showed a correlation between the presence of ROS and impaired sperm plasma membrane. Higher concentrations of reactive oxygen species are associated with a higher susceptibility to lipid peroxidation in sperm samples from less fertile Angus bulls, leading to mitochondrial dysfunction and damage to the sperm membrane, which in turn translates into worse conception rates [20].

Recently, extensive research has increasingly proved the key role of exosomes in the regulation of various complex reproductive processes [24–26]. Mitochondria are responsible for producing energy and regulating cellular metabolism, making them key components of overall cellular health [27]. Targeting activities to protect mitochondria can help regulate apoptosis (programmed cell death) and increase cell viability during various biological and technological processes. Recent studies have shown that EVs derived from brown adipose tissue improve mitochondrial function, promote follicle survival, improve fertility, and extend ovarian lifespan in ageing mice [28].

In this study, significantly lower (P <0.01) values of the mitochondrial membrane potential were observed in the control group (without EVs) compared to the group in which the spermatozoa were frozen in an extender containing 1.5 mg/ml EVs. The exosome content of the exosomes contributed to a better protection of cells against oxidative stress and could have a positive effect on obtaining the REDOX balance in the tested semen groups. This hypothesis is confirmed by the results of enzymatic activity presented in this study. In the groups containing 1.5 and 2.25 mg/ml EVs, significantly better parameters were obtained in terms of oxidative potential in terms of CAT and MDA. However, there were no significant differences (P<0.01) between the study groups in terms of SOD activity. Nevertheless, the tendency of this parameter was upward.

In the studies by Wang et al. [7], it was proved that the potential of the mitochondrial membrane potential of spermatozoa is positively correlated with sperm viability, increasing their fertilisation potential. In the experiment designed by us, it was observed that the tested groups (0.75; 1.5 and 2.25 ml / ml EVs) were characterised by both a higher mitochondrial membrane potential and viability compared to the frozen group without supplementation with EV. Sperm viability was the highest (92.6%) in the group containing 1.5 mg/ml EVs and was characterized by the highest percentage of HMP (27.65%).

Analysing sperm DNA integrity, no significant (P<0.01) differences (P <0.01) were found between the groups. However, it should be noted that the addition of EVs had a positive effect on the sperm groups of sperm. In this study, no relationship was found between sperm DNA structures and their viability or mitochondrial membrane. The percentage of cells damaged was very low even in the control group, which may confirm the effective protection of the extender used in the study. Many studies confirm that DNA damage reduces their fertilisation potential [29]. It has also been proven that a high level of DNA damage is associated with a lower rate of embryo development and more frequent absorption and miscarriages [30].

Increased ROS production and decreased antioxidant enzyme activity in sperm may trigger apoptotic pathways, resulting in decreased sperm motility [31]. The high sensitivity of mitochondrial membranes to low temperature can lead to ROS formation of ROS [32]. ROS generation may also be caused by activation of the mitochondrial apoptotic pathway and electron leakage from the electron transport chain [33, 34]. Changes in mitochondrial membrane fluidity may be another reason for ROS release [32]. In addition, mitochondrial membranes contain abundant polyunsaturated fatty acids (PUFAs) as preferred substrates for ROS. This leads to lipid peroxidation and the production of reactive lipid aldehydes that covalently bind to the electron transport chain and enhance ROS production in mitochondria [27, 35].

Research on the impact of extracellular vesicles (EVs) on sperm quality could have several implications for reproductive biology and cryopreservation techniques. They can be considered in several areas: 1) improved understanding of sperm function: Understanding how EVs influence sperm quality could lead to insight into various aspects of sperm biology, such as motility, morphology, and fertilisation potential. This knowledge could help in diagnosing and treating male infertility issues more effectively; 2) development of diagnostic tools: EVs derived from sperm or reproductive tissues could serve as biomarkers to assess sperm quality and diagnosing fertility problems. Analysing the composition and characteristics of these EVs could provide valuable information on the reproductive health of an individual; 3) enhancement of assisted reproductive technologies (ART): Incorporating knowledge about EVs into ART procedures, such as in vitro fertilisation (IVF) or intracytoplasmic sperm injection (ICSI), could improve success rates by selecting healthier sperm for fertilization. EV-based biomarkers could also aid in the selection of viable embryos during preimplantation genetic testing; 4) potential for therapeutic interventions: Manipulation of EVs or their cargo could offer new avenues to treat male infertility. For example, delivering specific molecules or factors through EVs to dysfunctional spermatozoa could potentially improve their quality and fertility potential; 5) impact on cryopreservation techniques: Cryopreservation is a crucial aspect of assisted reproduction, allowing the storage of sperm, eggs, or embryos for future use. Understanding how EVs are affected by cryopreservation and how they influence sperm viability post-thaw could lead to improvements in cryopreservation protocols, increasing the success rates of fertility treatments involving frozen sperm; 6) insights into reproductive pathologies: Research on EVs may also shed light on the mechanisms underlying reproductive disorders and diseases affecting male fertility. By elucidating how EVs contribute to these conditions, researchers may identify new therapeutic targets or diagnostic markers; 7) ethical considerations: As with any advancements in reproductive biology, ethical considerations surrounding the manipulation and use of EVs in fertility treatments will need to be addressed. This includes concerns related to informed consent, equitable access to treatments, and the potential long-term implications of altering reproductive processes.

## Conclusion

So far, little has been known about the role of EVs in maintaining the fertilisation potential of sperm. For the first time, the isolation of these vesicles and their use in an environment intended for cryopreservation of sperm cells were carried out. A comprehensive analysis of the protective properties on the structure and biochemistry of sperm was carried out. This study demonstrated a significant increase in cryotolerance of cryopreserved cells in extender supplemented with 1.5 mg/ml EVs. Exosomes isolated from the sperm plasma and supplemented in a concentrated dose in the extender for sperm freezing were shown to significantly improve cell cryostability by supporting the potentials of the mitochondrial membrane and protecting the cytoplasmic membrane of sperm.

Overall, research on the impact of EVs on sperm quality holds promise to advance our understanding of male fertility and improve reproductive health outcomes through diagnostic and therapeutic applications.

## Supporting information

S1 Fig Mitochondrial membrane potential. (XLSX)

S2 Fig DNA integrity and viability. (XLSX)

S3 Fig SOD, CAT, MDA activity. (XLSX)
